# On the transmission pattern of Kyasanur Forest disease (KFD) in India

**DOI:** 10.1186/s40249-015-0066-9

**Published:** 2015-08-19

**Authors:** Manoj V. Murhekar, Gudadappa S. Kasabi, Sanjay M. Mehendale, Devendra T. Mourya, Pragya D. Yadav, Babasaheb V. Tandale

**Affiliations:** National Institute of Epidemiology, Indian Council of Medical Research (ICMR), Chennai, Tamil Nadu India; Department of Health and Family Welfare, Shimoga, India; National Institute of Virology, ICMR, Pune, India

**Keywords:** Kyasanur Forest disease, Ticks, Virus disease, Disease outbreak, Transmission, India

## Abstract

Kyasanur Forest disease (KFD), a tick-borne viral hemorrhagic fever, is endemic in five districts of Karnataka state, India. Recent reports of the spread of disease to neighboring districts of the Western Ghats, namely Chamarajanagar district in Karnataka, Nilgiri district in Tamil Nadu, Wayanad and Malappuram districts in Kerala, and Pali village in Goa are a cause for concern. Besides vaccination of the affected population, establishing an event-based surveillance system for monkey deaths in the national parks, wildlife sanctuaries and reserve forests of the Western Ghats would help detect the disease early and thereby help implement appropriate control measures.

**Electronic supplementary material:**

The online version of this article (doi:10.1186/s40249-015-0066-9) contains supplementary material, which is available to authorized users.

## Multilingual abstracts

Please see Additional file 1 for translations of the abstract into the six official working languages of the United Nations.

## Background

The Kyasanur Forest disease (KFD) was first detected in 1956 in Karnataka, India [1]. The KFD virus (KFDV) is maintained by ticks, mammals, and bird cycles. The black-faced langur (*Presbytis entellus*) and the red-faced bonnet monkey (*Macaca radiata*) act as sentinel animals, as they are very susceptible to KFDV, as humans are [2, 3]*.* Epizootics of this infection cause a heavy burden of mortality in these monkeys, which indicates likely outbreaks, mostly from December to June. Ticks drop as soon as the animal dies, thus generating hotspots for infection. The virulence of KFDV is noticeable; numerous infections were reported in laboratory and field personnel who were directly dealing with a KFD outbreak. It is a biphasic disease with an incubation period of three to eight days. Symptoms include a sudden onset of fever, headache, myalgia, gastrointestinal symptoms, and bleeding manifestations, which may occur three to four days after the onset of the illness. After one to two weeks, 80–90 % of patients recover without complications. In the remaining patients, a biphasic course with mild meningoencephalitis and fever develops [2, 3].

The KFDV was first isolated from sick monkeys captured in Kyasanur forest, Shimoga; hence its name. KFD is known for its unique existence in five districts - Shimoga, Uttar Kannada, Dakshina Kannada, Chikmaglur and Udupi of Karnataka state [2, 3]. Since better diagnostic methods have become available [4], KFD infection in ticks and/or monkeys, as well as sporadic cases in humans, have been reported from newer areas *i.e.* Chamarajanagar district in Karnataka, Nilgiri district in Tamil Nadu, Wayanad and Malappuram districts in Kerala, and Pali village in Goa. The affected districts are all part of the Western Ghats forest. People with occupational exposure to rural or outdoor settings (*e.g.*, hunters, herders, forest workers, farmers) in these districts are potentially at risk for infection if they come into contact with infected ticks.

The KFD is placed in the biosafety risk group-4 category as per the guidelines of the Center for Disease Control and Prevention [5]. The main gaps in understanding the epidemiology of this disease are: 1) determining whether KFD has the potential for person-to-person transmission and 2) detecting the presence of KFD in newer areas (see Table 1).

**Table 1:** Presence of KFD in other areas than the previously known five endemic districts in Karnataka, in the last three years

Year	KFD presence in India
2012	100 confirmed cases in Karnataka; tick and monkey positivity
2012	KFDV confirmed in monkey specimen in Nilgiri, Tamil Nadu
2012–13	Outbreak in the Bandipur National Tiger Reserve, Karnataka; confirmed by human and monkey positivity
2013	Human case confirmed in Wayanad, Kerala
2014	Outbreak in seven health centers in Thirthahalli, Shimoga, Karnataka
2014	Human case confirmed in Wayanad, Kerala
2014	Outbreak in a tribal population, Malappuram, Kerala
2014	Anti-KFD IgG antibody positivity in a tribal population of the Palakkad and Wayand districts, Kerala
2015	Confirmed in monkey specimen in Nilambur, Malappuram, Kerala
2015	Tick positivity for KFDV in Wayanad, Kerala
2015	Outbreak in Wayanad, Kerala [18 confirmed cases; Pulpally (7), Mullankolly (8), Chethayalayam (1), and Poothadi (2)]
2015	Outbreak in Shimoga, Karnataka [35 confirmed cases]
2015	Outbreak in Pali village, Sattari Taluka, northeast Goa [18 confirmed cases]

### A KFD outbreak in Malappuram, Kerala, 2014

We congratulate one of this paper’s authors, Dr. Tandale, and his colleagues, for their investigation of a KFD outbreak, and for identifying a new focus of virus activity, in the Malappuram district, in Kerala [6]. This is the fourth report of KFD activity beyond the five endemic districts – Shimoga, Uttar Kannada, Dakshina Kannada, Chikmagalur and Udupi. From November 2012 to May 2013, KFD outbreak was reported among the forest workers in the Bandipur Tiger Reserve in Chamarajanagar [7]. During the same period, KFD activity was detected in monkeys and/or ticks in Nilgiri and Wayanad [7]. Human cases of KFD were also reported in the tribes residing in the forest areas of Wayanad in 2013 and 2015 [8, 9]. Even more recently, KFD cases have been reported in the Pali village in Goa [10].

Tandale *et al.* report two clusters of KFD in Malappuram [6]. In the first cluster, one person out of a family of eight had serological evidence of infection, while the second cluster had four secondary cases (1 RT-PCR positive) and one of the 17 contacts had serological evidence of a recent infection. The term ‘secondary case’ indicates a case infected by a primary case. The data presented in Table 1 of the reference paper (6) gives the impression that the secondary cases/infections in the two clusters have resulted from primary cases; which could be because of person-to-person transmission. However, KFD is transmitted primarily through tick bites and there is no reported evidence of further transmission from the cases. The secondary cases/infections in the two clusters could in fact be due to tick bites because of their similar environmental exposure with primary cases.

### Is there evidence of human-to-human transmission?

With evidence of new emerging cases and diagnosis from other states in India, the question that begets answering why is the disease spreading to these new areas? Is human-to-human transmission occurring? Earlier published reports confirmed that a large number of laboratory-associated infections (LAIs) [2] and cases were observed in personnel who worked in the affected forest. There was a limited understanding and awareness about biosafety in the early days as compared to recent years. The data in our current research [6] doesn’t indicate anything about person-to-person transmission. The term ‘secondary case’ is used to indicate timing of case onset in family members and neighborhoods following the first case (see Table 1, [6]). Transmission is most likely occurring due to tick bites, at various times, among the tribal community that lives in the affected forest.

The disease has been known for more than six decades, but no hospital has ever reported any nosocomial KFD infections or cases, which occurred between close human contacts. No clustering of cases that suggest person-to-person transmission has ever been seen. States such as Kerala, Tamil Nadu, and Goa reported cases occurring in areas surrounding the forest where there is a wide spread presence of the *Haemaphysalis* vector tick. In these areas, close contact with animals that graze in the affected forest might transport the infected ticks to villages and thus increase the probability of tick bites to their inhabitants. In such situations, a small clustering—where there might be more than one case in a household infected by tick bites–might be observed.

We observed a similar phenomenon in 2014 in an outbreak in the Thirthahalli area, where more than 100 cases were recorded amongst villagers, many of those never visiting the forest [11].

Transovarial and transstadial transmission of KFDV in ticks is important for its maintenance in a natural cycle. Small mammals further help in spreading the virus. KFDV is not detected in body secretions except blood and hence nosocomial infections or human-to-human transmission is unlikely. In KFD cases, typical hemorrhages are also not manifested, but in the majority of severe cases, there are reports of gastrointestinal bleeding. Humans are considered as dead-end hosts because ticks infests only animals. Humans get infected due to accidental bites by infected nymphs.

Earlier records show a presence of antibodies to KFD in animals in the semi- arid Kutch district and the Andaman Islands [3, 12]. No adequate attempts have been made to look for the presence of the virus in other parts of India. So far, known hotspots are mostly in the Western Ghats region, especially in Karnataka, however, the Ghats also stretches to other states. It is likely that KFD might have spread to some of the adjoining regions of the Western Ghats. Presence of this virus in a human patient has also been reported in China, close to the Indian border [13]. Prior to 2012, modern diagnostic tests such as PCR and ELISA technology were not available to test for this disease [4]. But now, due to the availability of these tests and increased awareness, more and more suspected cases from other areas are being screened for KFD. Thus, the presence of this virus in many previously unknown KFD areas has come into light.

The presence of KFDV that has been reported in China showed close genetic similarity with the Indian KFD strains [13]. Alkhurma, a variant or subgroup virus, has been reported in Saudi Arabia. There is a common ancestry between KFDVs in India and Saudi Arabia, despite their large geographic separation, indicating a wide movement of the virus, possibly carried by birds [14, 15].

### Future research priorities

The spread of KFD to newer areas is a major cause for concern. The five KFD endemic districts in Karnataka, as well as Chamarajanagar, Nilgiri, Wayanad, and Malappuram districts of the Western Ghats, which, stretch from north to south, covers the states of Gujarat, Maharashtra, Goa, Karnataka, Tamil Nadu, and Kerala, and comprises 39 properties including wildlife sanctuaries, national parks, and reserve forests [16]. The spread of KFDV to newer areas is most probably due to the movement of monkeys and small rodents, which harbor the virus [2, 3]. People residing in the forest areas, as well as those working in the parks, sanctuaries, and reserve forests remain at risk of acquiring the disease. As the presence of the disease often becomes noticeable when enzootic infections occur and sentinel animals such as monkeys start dying [2, 3], establishing an event-based surveillance system for monkey deaths in the national parks, wildlife sanctuaries, and reserve forests of the Western Ghats, as well as neighboring villages. This would help detect the disease early and thereby help institute appropriate control measures. Conducting serosurveys in different districts of the region would also help in the mapping of the disease.

Since 1990, formalin inactivated tissue culture vaccine has been used to control KFD in the five endemic districts of Karnataka (Directorate of Health and Family Welfare Services, Government of Karnataka, Manual on Kyasanur Forest disease, 2005, unpub. data). The vaccination strategy involves mass vaccination programme during the months of August to November in the areas that have reported KFD activity (defined as laboratory evidence of confirmed cases in monkeys/humans or infected ticks), as well as surrounding villages within a radius of five kilometers. Two doses of the vaccine are administered to individuals aged 7–65 years with an interval of one month. The immunity acquired after the vaccination is short lived and hence annual boosters are recommended for five consecutive years after the last confirmed case in the area. However, the current vaccine is not very effective [17–19]. The coverage of the vaccine in the five endemic districts was also found to be low, thus indicating a low acceptance of the vaccine [17, 19]. Full genome analyses of the current KFDV strains could determine how effective the current vaccine is, and detailed studies on the genomes of the KFDV strains from different geographical areas could indicate the level of protection the vaccine could have.

Other future research priorities for the control of KFD include understanding reasons for the vaccine’s low coverage, studying the long-term protection offered by booster doses, and evaluating the appropriateness of the vaccination strategy. Future research also needs to focus on further refining what constitutes a vaccine candidate in order to make the vaccine more effective and avoid the need for periodic boosters [17–19].

## Additional file

Additional file 1:
**Multilingual abstracts in the six official working languages of the United Nations.** (PDF 282 kb)

## Abbreviations

KFD: Kyasanur forest disease
KFDV: Kyasanur forest disease virus
RT-PCR: Reverse transcription polymerase chain reaction
